# Genetic Structure of the Tiger Mosquito, *Aedes albopictus*, in Cameroon (Central Africa)

**DOI:** 10.1371/journal.pone.0020257

**Published:** 2011-05-24

**Authors:** Basile Kamgang, Cécile Brengues, Didier Fontenille, Flobert Njiokou, Frédéric Simard, Christophe Paupy

**Affiliations:** 1 Unité mixte de recherche MIVEGEC (UM1-CNRS 5290-IRD 224), Institut de Recherche pour le Développement (IRD), Montpellier, France; 2 Laboratoire de Recherche sur le Paludisme, Organisation de Coordination pour la lutte contre les Endémies en Afrique Centrale (OCEAC), Yaoundé, Cameroon; 3 Faculté des Sciences, Université de Yaoundé I, Yaoundé, Cameroon; 4 Institut de Recherche en Sciences de la Santé (IRSS), Bobo-Dioulasso, Burkina-Faso; 5 Unité de Recherche en Ecologie de la Santé, Centre International de Recherches Médicales de Franceville (CIRMF), Franceville, Gabon; Universidade Federal do Rio de Janeiro, Brazil

## Abstract

**Background:**

*Aedes albopictus* (Skuse, 1884) (*Diptera*: *Culicidae*), a mosquito native to Asia, has recently invaded all five continents. In Central Africa it was first reported in the early 2000s, and has since been implicated in the emergence of arboviruses such as dengue and chikungunya in this region. Recent genetic studies of invasive species have shown that multiple introductions are a key factor for successful expansion in new areas. As a result, phenotypic characters such as vector competence and insecticide susceptibility may vary within invasive pest species, potentially affecting vector efficiency and pest management. Here we assessed the genetic variability and population genetics of *Ae. albopictus* isolates in Cameroon (Central Africa), thereby deducing their likely geographic origin.

**Methods and Results:**

Mosquitoes were sampled in 2007 in 12 localities in southern Cameroon and analyzed for polymorphism at six microsatellite loci and in two mitochondrial DNA regions (ND5 and COI). All the microsatellite markers were successfully amplified and were polymorphic, showing moderate genetic structureamong geographic populations (*F_ST_* = 0.068, P<0.0001). Analysis of mtDNA sequences revealed four haplotypes each for the COI and ND5 genes, with a dominant haplotype shared by all Cameroonian samples. The weak genetic variation estimated from the mtDNA genes is consistent with the recent arrival of *Ae. albopictus* in Cameroon. Phylogeographic analysis based on COI polymorphism indicated that *Ae. albopictus* populations from Cameroon are related to tropical rather than temperate or subtropical outgroups.

**Conclusion:**

The moderate genetic diversity observed among Cameroonian *Ae. albopictus* isolates is in keeping with recent introduction and spread in this country. The genetic structure of natural populations points to multiple introductions from tropical regions.

## Introduction

The Asian “tiger” mosquito, *Aedes* (Stegomyia) *albopictus* (Skuse, 1894) (Diptera: Culicidae), is a major vector of dengue (DENV, Flaviriridae, flavivirus) and chikungunya (CHIKV, Togaviridae, alphavirus) viruses in tropical areas [1]–[3]. This mosquito species originated in southeast Asia [4], and has rapidly invaded numerous countries in Africa, the Middle East, Europe and the Americas over the last three decades [5]. This rapid global spread was favored by international trade, especially in used tires [6]. In Africa, *Aedes albopictus* was first reported in South Africa in 1989, where it was immediately controlled [7], and two years later in Nigeria, where it pullulated [8]. In Central Africa, *Ae. albopictus* was first recorded in Cameroon in 2000 [9], followed by rapid expansion in most of the south [10]. *Aedes albopictus* is now the dominant species at the periphery of urban centers such as Yaoundé and Douala [11], where it has gradually replaced the resident species, *Aedes aegypti* (L., 1762).

Coinciding with the spread of *Ae. albopictus*, numerous DENV and CHIKV epidemics occurred in Cameroon [12] and Gabon [13] during the 2000s. *Ae. albopictus* was incriminated as the main vector of both viruses during a 2007 dual outbreak in Libreville, Gabon [3], [13]. In addition, *Ae. albopictus* isolates in Cameroon proved to be orally susceptible to DEN-2 virus and highly competent for CHIKV [3].

*Aedes albopictus* exhibits strong physiological and ecological plasticity, allowing it to thrive in a wide range of climates and habitats. Known as a sylvatic species in Asia [4], *Ae. albopictus* has now adapted to human environments and is preferentially found in suburban areas, although it has also been recorded in densely populated urban areas such as Rome, Italy [14]. The range of larval development sites suitable for *Ae. albopictus* is extremely broad and includes natural water collections (e.g., bamboo stumps, bromeliads and tree holes) and artificial containers (e.g., water storage containers, abandoned car parts and all other discarded items where rainwater can accumulate) [15]. The mosquito preferentially feeds on mammals [15], but females can feed on most groups of cold- to warm-blooded vertebrates, including reptiles, birds and amphibians [16]. The feeding preferences of *Ae. albopictus* vary with the geographic origin of local mosquito populations [17]. This ability of *Ae. albopictus* to feed on different animal species enhances not only its biological traits (e.g., fecundity and survival) but also its capacity to propagate zoonotic pathogens across animal species and to humans [18]. Finally, the broad host spectrum would facilitate invasion of diverse environments ranging from forests to urban areas.

Several phylogeographic studies have been undertaken to determine the origin of invasive *Ae. albopictus* populations. An early study based on isoenzymatic markers suggested that North and South American populations (USA and Brazil) were closely related to Japanese populations [19]. This was confirmed by a study suggesting that “temperate populations” in the USA, Japan and Italy form a genetic cluster distinct from tropical Asian populations [20]. More recently, however, studies of mitochondrial markers challenged the common origin of North and South American populations [21], and suggested that Brazilian populations were related to Southeast Asian populations rather than to temperate Asian populations [22]. Differences in biological traits, such as the winter diapause exhibited by USA populations but not by Brazilian populations [15], support this hypothesis. It has also been shown that the vector competence of *Ae. albopictus* varies according to its geographic origin [23]–[25]. As biological traits of *Ae. albopictus* are genetically determined, and as these traits may influence virus transmission in newly colonized areas, it is important to determine the geographic origin of invading populations.

As little is known of the geographic origin or genetics of *Ae. albopictus* in Africa, we undertook a genetic study of *Ae. albopictus* in Cameroon.

## Materials and Methods

### Ethics Statement

An institutional clearance for this study, including the sampling of mosquito, was granted by Health Ministry of Cameroon.

### Mosquito sampling

**Figure 1:**
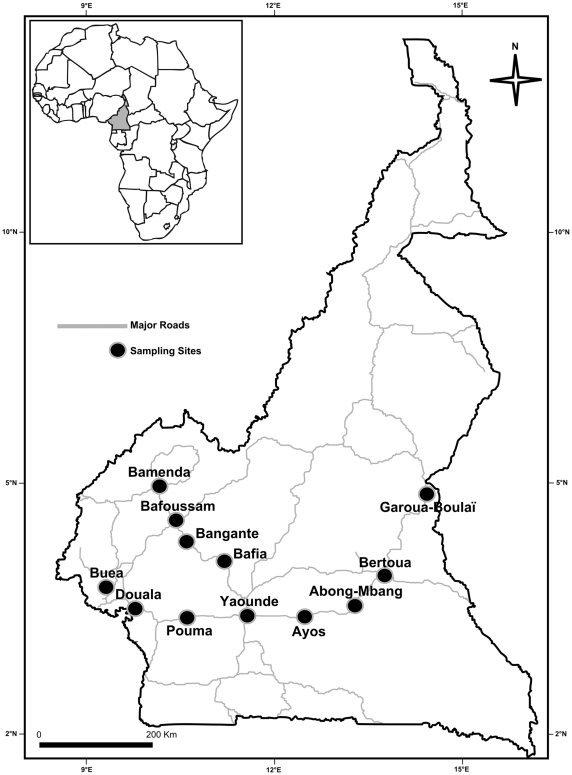
Location of *Ae. albopictus* sampling sites in Cameroon, April 2007.

*Aedes albopictus* mosquitoes were sampled as larvae or pupae in 12 localities in South Cameroon during April 2007. The geographic locations and main characteristics of the sampling sites are summarized in Figure 1 and Table 1. Aquatic stages (field generation, F0) were collected from domestic containers (tanks) and peri-domestic water collections (used tires, discarded containers, etc.) and forwarded in plastic boxes to insectaries. Each sample from a given locality consisted of pooled larvae from 4–6 breeding places, to avoid inbreeding. In the insectaries, mosquito larvae and pupae were transferred to pans containing water for rearing to adulthood. Adults were examined morphologically to confirm they were *Ae. albopictus* and were then stored in individual tubes containing a desiccant at −20°C until molecular analysis.

**Table 1:** Characteristics of *Ae. albopictus* sampling sites in Cameroon, April 2007.

Region	Locality	Number of sampled larval siteshabitat	Type of larval habitat	Geographic coordinate	Climate
**East**	**Abong-Mbang**	5	Used tires	04°00′N; 13°19′E	Subequatorial Guinean climate
	**Bertoua**	5	Tin cans, used tires	04°33′N; 13°46′E	Subequatorial Guinean climate
	**Garoua-Boulai**	5	Discarded tanks, tree holes	05°50′N; 14°28′E	Subequatorial Guinean climate
**Center**	**Ayos**	4	Used tires	03°52′N; 12°29′E	Subequatorial Guinean climate
	**Bafia**	5	Tin cans, tree holes, used tires	04°44′N; 11°11′E	Subequatorial Guinean climate
	**Yaoundé**	6	Car wrecks, used tires	03°54′N; 11°37′E	Subequatorial Guinean climate
**South-West and Littoral**	**Buea**	4	Drums, used tires	04°09′N; 09°13′E	Equatorial monsoon with heavy summer rains
	**Douala**	5	Tarpaulins, used tires	04°02′N; 09°41′E	Equatorial monsoon with heavy summer rains
	**Pouma**	4	Discarded tanks, tin cans	03°50′N; 10°33′E	Equatorial monsoon with heavy summer rains
**West and North-West**	**Bamenda**	5	Car wrecks, tin cans, used tires	05°58′N; 10°13′E	Equatorial monsoon (highlands)
	**Banganté**	4	Drums, tin cans	05°09′N; 10°33′E	Equatorial monsoon (highlands)
	**Bafoussam**	5	Car wrecks, tin cans, used tires	05°29′N; 10°26′E	Equatorial monsoon (highlands)

### Microsatellite analysis

Total DNA was extracted from whole mosquito bodies, as described by Morlais et al. [26]. DNA pellets were resuspended in sterile water and stored at −20°C. Genetic polymorphism was assessed at six microsatellite markers: AealbA9, AealbB6, AealbB51, AealbB52, AealbD2 and AealbF3 [27].

DNA was amplified in a GeneAmp 9600 thermal cycler (PerkinElmer, USA) in 25-µl reaction mixes containing 2 µl of 1/5-diluted total DNA, 2.5 µl of 10× reaction buffer (Qiagen, USA), 0.5 mM MgCl_2_ (for AealbB6 and AealbD2), 0.2 mM each dNTP (Eurogentec, Belgium), 10 pmol of each primer, and 0.5 U of Taq polymerase (Qiagen, USA). The 5′ end of the forward primer was labeled with a fluorescent dye (FAM, NED, PET and VIC). Cycling conditions were as follows: 5 min at 94°C, followed by 35 cycles of 45 s at 94°C, 30 s at the annealing temperature (TA), 45 s at 72°C, and 30 min at 72°C. PCR products were diluted in water (1/50 to 1/200, according to the sensitivity of each marker) and pooled with other compatible products according to allele size range and fluorescent dye. Pools were prepared by adding 0.4 µl of GS 500 Liz internal size standard™ (Applied Biosystems, USA) and HD formamide to 1 µl of each diluted amplification product, for a total volume of 20 µl. The mixture was heated at 95°C for 3 minutes before migration in an ABI Prism™ 3130 XL automatic sequencer (Applied Biosystems). Microsatellite alleles were scored with GeneMapper software (Applied Biosystems).

Genetic diversity per locus and per sample was characterized by estimating allele richness, *Rs* [28], and unbiased expected heterozygosity *H_e_* [29], using FSTAT2.9.3.2 [30] and GENETIX4.05.2 [31]. The frequency of null alleles at each locus within each population, linkage disequilibrium between pairs of loci, deviations from Hardy-Weinberg equilibrium (HWE), and genetic differentiation indices were estimated with GENEPOP4.07 [32]. *F_IS_* and *F_ST_* estimates were calculated as described in [33], and were tested for statistical significance with exact tests available in GENEPOP4.07. The overall significance of multiple tests was estimated with Fisher's combined probability test. Critical significance levels for multiple testing were corrected with the sequential Bonferroni procedure [34]. The frequency of null alleles at each locus within each population was determined with GENEPOP V4.07, and the allele and genotype frequencies were then adjusted accordingly in MICROCHECKER V2.2.3 [35]. The null allele adjusted dataset was compared to the original dataset to explore the effect of null alleles on estimates of genetic differentiation.

Isolation by distance (IBD) was estimated with Mantel's test in GENEPOP4.0.7, using the correlation between genetic and geographic distances by the regression of pairwise F_ST_/(1−F_ST_) on the natural logarithm (Ln) of straight-line geographic distance [36]. UPGMA trees were computed with POWERMARKER 3.25 [37], based on microsatellite Nei's genetic distance [38]. The robustness of population trees was evaluated by bootstrapping over loci. One thousand bootstrapped trees were summarized to obtain a consensus tree, using the CONSENS module in the Phylip package [39], followed by editing with TREEVIEW [40].

A Bayesian approach available in STRUCTURE 2.2 was used to infer the number of clusters (*K*) in the data set, without prior information of the sampling locations [41]. A model in which allele frequencies correlated within populations was assumed (λ was set at 1, the default value). The software was run with the option of admixture, allowing for some mixed ancestry within individuals, and α was allowed to vary. We used 20 independent runs for each value of *K* (K = 1 to 12), with a burn-in period of 100 000 iterations and 400 000 replications. The method of Evanno et al. [42] was used to determine the most likely number of clusters. This approach uses an *ad hoc* quantity, Δ*K*, based on the second-order rate of change of the likelihood function between successive values of *K*.

### Mitochondrial DNA analysis

At least ten individuals per locality, selected among those previously analyzed for microsatellite markers, were used to explore sequence polymorphism in the mitochondrial genes *ND5* and *COI*. DNA samples were used as templates to amplify a 400-bp fragment of *ND5* and a 550-bp fragment of *COI*. Two sets of primers were used: 5′TCCTTAGAATAAAATCCCGC-3′(FOR-ND5) and 5′-GTTTCTGCTTTAGTTCATTCTTC-3′ (REV-ND5) for ND5 [21], and 5′-GGAGGATTTGGAAATTGATTAGTTC-3′ (FOR-COI) and 5′-CCCGGTAAAATTAAAATATAAACTTC-3′ (REV-COI) for COI [43]. PCR amplification used a GeneAmp 9600 thermal cycler (PerkinElmer) with 21-µl reaction mixes containing 4 µl of 1/50-diluted template DNA, 2.5 µl of 10× reaction buffer (Qiagen), 0.5 mM MgCl_2_ (for ND5), 0.2 mM dNTP, 10 pmol of each primer and 0.5 U of Taq polymerase (Qiagen). The thermal cycling conditions consisted of 3 min at 94°C, 35 cycles of 30 s at 94°C, 45 s at TA (52°C for *ND5* and 54°C for *COI*), and 45 s at 72°C, followed by 10 min at 72°C. PCR products were detected by agarose gel electrophoresis in Tris-borate-EDTA buffer (TBE), stained with 0.5 µg/ml ethidium bromide in TBE, and visualized over UV light. PCR products were purified with the Ampure kit (Beckman Coulter, France, Paris) and sequenced on both strands using ABI Prism BigDye Terminator version 3.1 (Applied Biosystems). Each 10-µl sequencing reaction mix contained 1 µl of Ready Reaction mix (Applied Biosystems), 1 µl of 5× sequencing buffer, 3.2 pmol/µl primer, and 0.5–1.0 µl of purified PCR product. The reaction consisted of an initial denaturation step of 1 min at 96°C, followed by 25 cycles of 10 s at 96°C, 5 s at 50°C, and 4 min at 60°C, with a final elongation step of 3 min at 72°C. Sequencing reaction products were purified with the Agencourt CleanSeq kit (Beckman Coulter) and analyzed on an ABI 3130XL automatic sequencer (Applied Biosystem).

Sequences were analyzed with SEQSCAPE software 2.5 (Applied Biosytems) and aligned with Clustal W [44]. *ND5* and *COI* sequences were numbered according to the reference sequences (GeneBank ID AJ971026.1 and AJ971015.1, respectively). Basic sequence statistics, including the number of haplotypes per sample (Hp), the number of segregating sites (S), haplotype diversity (HpD), nucleotide diversity (π) and the average number of nucleotide differences were computed with DnaSP 4.10.9 [45]. Neutrality tests used the statistics of Tajima [46], Fu and Li [47] and Fu [48] in DnaSP 4.10.9. The phylogenetic relationships between COI and ND5 haplotypes recorded in Cameroon and previously published sequences of *Ae. albopictus* from Asia, the Americas, the Indian Ocean and Europe were explored with Bayesian inference analysis. MrModeltest v2.2 [49] was first used to select the model best fitting the *COI* and *ND5* nucleotide sequence data (under Akaike's information criterion). Analyses were performed with MrBayes 3.1.2 [50] and four Markov chains were run for 2 000 000 generations (sampling every 100 generations) to allow adequate time for convergence. The first 2500 trees were discarded as burn-in, and the remaining 17 500 sampled trees were used to estimate the 50% majority rule consensus tree and the Bayesian posterior probabilities. All MCMC runs were repeated twice to confirm consistent approximation of the posterior parameter distributions.

## Results

### Microsatellite analysis

#### Genetic variability and Hardy Weinberg expectations

A total of 452 *Ae. albopictus* specimens collected in 12 localities in Cameroon were successfully genotyped at all 6 microsatellite loci (Table 2). All loci were polymorphic, showing a number of distinct alleles ranging from 2 at locus AaelbB51 to 17 at loci AaelbA9 and AaelbB6. However, at AaelbB51, the dominant and shared allele across all samples was fixed in Garoua-Boulai, Abong-Mbang, Buea, Bamenda and Bafoussam. Over all the loci, the diversity indexes (*Rs* and *He*) were lowest in Bafoussam and highest in Buéa (Table 2). Departures from Hardy-Weinberg equilibrium associated with significant heterozygote frequency deficits were detected in 7 of 67 tests that could be conducted (one in AaebB51 and six in AaelbB6, see Table 2). All these deviations were attributed to the presence of null alleles, with frequencies of unobserved alleles ranging from 0.013 to 0.274 in loci with significant heterozygote deficiency (see Table S1). However, null alleles did not significantly bias our interpretation, as re-analysis of adjusted datasets gave similar results. Significant linkage disequilibrium was revealed in three occasions (AaelbA9-AaelbB6 and AaelbA9-AaelbD2 in Bamenda, and AaelbB6-AaelbD2 in Bertoua).

**Table 2:** Genetic variability of *Ae. albopictus* populations surveyed in Cameroon, based on six microsatellite loci.

	AaelbB6			AaelbD2			AaelbA9			AaelbB51			AaelbB52			AaelbF3			All loci		
Sample (N)	*N_all_*	*R_s_*	*H_e_*	*N_all_*	*R_s_*	*H_e_*	*N_all_*	*R_s_*	*H_e_*	*N_all_*	*R_s_*	*H_e_*	*N_all_*	*R_s_*	*H_e_*	*N_all_*	*R_s_*	*H_e_*	*N_all_*	*R_s_*	*H_e_*
**Abong-Mbang (40)**	9	6.42	**0.55**	9	5.10	0.60	11	8.97	0.88	1	1.00	-	3	1.95	0.08	4	3.33	0.50	6.2	4.46	0.43
**Bertoua (40)**	9	5.28	**0.44**	7	5.54	0.67	12	8.81	0.82	2	1.74	0.07	3	2.88	0.31	3	2.93	0.47	6.0	4.53	0.46
**Garoua-Boulai (27)**	5	4.21	0.32	7	6.35	0.81	9	7.33	0.68	1	1.00	-	2	2.00	0.39	3	0.50	0.50	4.5	3.91	0.45
**Ayos (40)**	4	3.27	**0.27**	7	5.38	0.62	11	9.31	0.86	2	1.44	0.03	3	2.63	0.36	4	3.36	0.54	5.2	4.23	0.45
**Bafia (40)**	9	6.83	**0.74**	7	5.93	0.66	9	7.49	0.82	2	1.73	0.07	2	1.58	0.05	3	2.35	0.45	5.3	4.32	0.47
**Yaoundé (40)**	8	5.23	0.56	8	6.21	0.75	10	8.12	0.78	2	1.58	0.05	4	3.24	0.31	3	2.84	0.36	5.8	4.53	0.47
**Buea (25)**	6	6.00	0.69	6	5.47	0.73	11	9.56	0.84	1	1.00	-	4	3.58	0.32	4	3.51	0.57	5.3	4.85	0.53
**Douala (40)**	8	6.04	0.76	8	6.72	0.79	8	6.84	0.69	2	1.74	0.07	3	2.10	0.1	3	2.84	0.48	5.3	4.38	0.48
**Pouma (40)**	6	3.98	0.27	6	5.30	0.77	11	7.98	0.74	2	1.93	**0.14**	4	3.26	0.33	4	3.24	0.53	5.5	4.28	0.46
**Bamenda (40)**	8	6.14	**0.61**	5	4.58	0.72	11	7.88	0.74	1	1.00	-	2	2.00	0.25	3	2.77	0.53	5.0	4.06	0.47
**Banganté (40)**	7	5.73	**0.50**	5	4.31	0.68	10	8.30	0.86	2	1.83	0.1	2	1.98	0.20	3	2.58	0.50	4.8	4.12	0.47
**Bafoussam (40)**	5	4.65	**0.49**	3	3.00	0.61	5	4.48	0.76	1	1.00	-	2	2.00	0.48	2	2.00	0.49	3.0	2.85	0.46
**All samples (452)**	17	7.09	**0.52**	11	5.97	0.70	17	9.52	0.79	2	1.53	**0.05**	4	2.79	**0.27**	5	3.32	0.54	9.3	5.04	**0.48**

N, sample size (number of mosquitoes); *N_all_*, number of alleles; *R_s_*, allele richness [28]; *H_e_*, Nei's unbiased estimate of expected heterozygosity [29]; Values in bold highlight significant deficit in heterozygote (*P*<0.05 after Bonferroni correction).

#### Genetic differentiation

**Figure 2:**
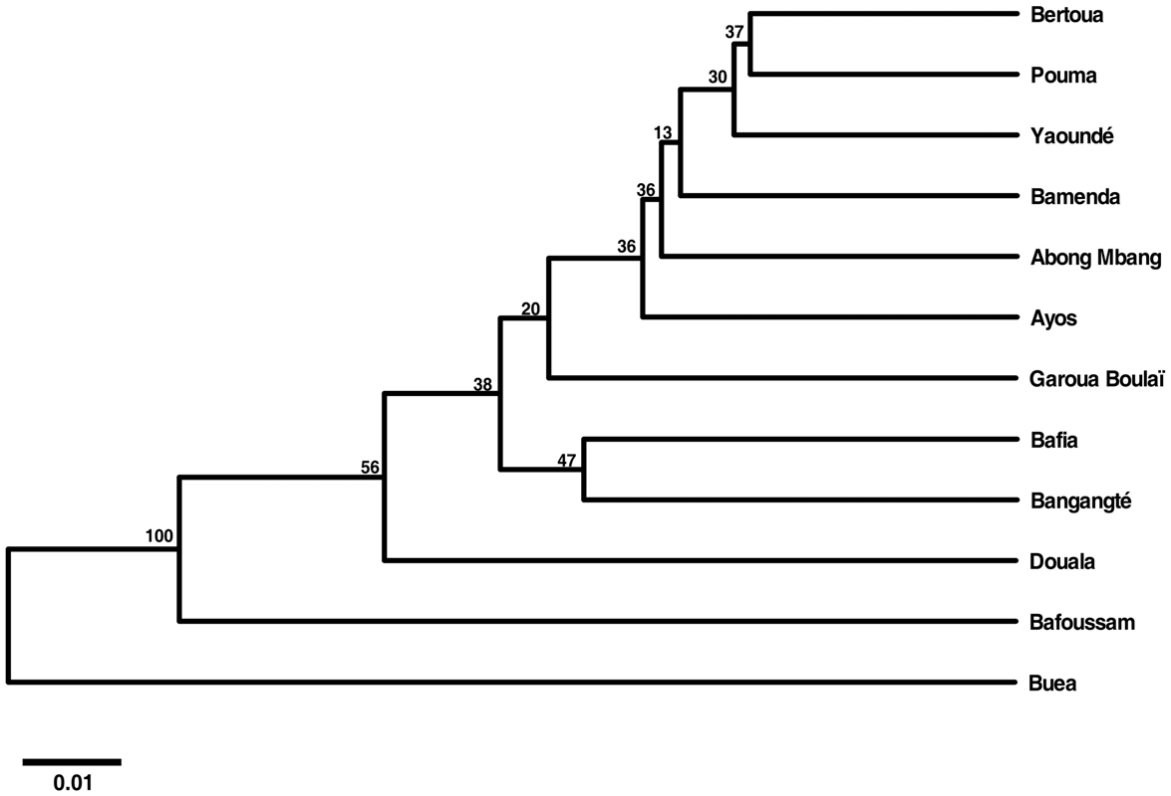
Dendrogram based on microsatellite Nei's genetic distance [**37**] clustering by UPGMA methods. The genetic relationship among 12 *Ae. albopictus* populations sampled in Cameroon is shown.

Overall genetic differentiation between samples was moderate and statistically significant (F_ST_ = 0.068, P<0.0001). Pairwise analysis of populations indicated significant genetic differentiation for most sample pairs (61/66), associated with F_ST_ estimates ranging from 0.006 (Bertoua-Abong-Mbang) to 0.176 (Buea-Yaounde) (Table 3). The UPGMA unrooted tree constructed with pairwise genetic distances and bootstrap values supporting node clearly indicated that the Buea sample was genetically distant from all the other samples (Figure 2). The topology of the tree shown in Figure 2 suggested that the pattern of genetic differentiation was not shaped by the geographic distance between sampling sites, in agreement with Mantel's test (*P* = 0.33). Bayesian cluster analysis identified 2 distinct genetic clusters in the dataset, although most of geographic populations appeared as a mixture of specimens from the two clusters except Buea and Garoua-Boulaï that appear made up of a single dominant cluster (see Figure S1).

**Table 3:** Matrix of pairwise estimates of *F_ST_* among *Ae. albopictus* populations from Cameroon.

Sample	Abong-Mbang	Bertoua	Garoua-Boulai	Ayos	Bafia	Yaoundé	Buea	Douala	Pouma	Bamenda	Banganté
**Abong-Mbang**	**-**										
**Bertoua**	0.006	**-**									
**Garoua-Boulai**	0.083***	0.063***	**-**								
**Ayos**	0.026*	0.017	0.059***	**-**							
**Bafia**	0.023***	0.042***	0.100***	0.063***	**-**						
**Yaoundé**	0.026*	0.021*	0.039***	0.045***	0.053***	**-**					
**Buea**	0.144***	0.141***	0.163***	0.162***	0.164***	0.176***	**-**				
**Douala**	0.073***	0.073***	0.142***	0.099***	0.072***	0.075***	0.159***	**-**			
**Pouma**	0.039***	0.023	0.013*	0.039	0.069***	0.018	0.140***	0.101***	**-**		
**Bamenda**	0.036**	0.036**	0.023**	0.049***	0.047***	0.020*	0.118***	0.075***	0.016***	**-**	
**Banganté**	0.020***	0.026***	0.071***	0.029**	0.116***	0.051***	0.127***	0.086***	0.036***	0.038**	**-**
**Bafoussam**	0.089***	0.067***	0.101***	0.090***	0.045***	0.096***	0.162***	0.156***	0.077***	0.094***	0.054**

The statistical significance of *F_ST_* estimates was assessed using the Fisher exact test of homogeneity of genotypic frequencies [32]. In bold, significant P values (*P<0.05, **P<0.001, ***P<0.0001) after correction for multiple tests.

### Mitochondrial DNA analysis

Nucleotide sequences of mtDNA ND5 (400 bp) and COI (500 bp) were obtained for 153 *Ae. albopictus* specimens sampled at the 12 Cameroonian locations. Overall, the polymorphism in the ND5 region was low, with only three segregating sites defining four closely related haplotypes, resulting in low haplotype and nucleotide diversity indexes (Table 4 and 5). The most frequent haplotype, H1 (86.3%), was detected in all geographic samples. Similarly, low levels of polymorphism were detected in the COI gene (Table 4 and 5). The most frequent haplotype, H1 (86.9%), was found in all geographic samples. Tajima's D and Fu's Fs statistics were negative for both genes (D = −0.8950 and Fs = −1.827 for ND5; D = −0.347 and Fs = −0.558 for COI), but not statistically significant. Negative values of these statistics might reflect either a selective sweep or recent demographic expansion [46].

**Table 4:** MtDNA COI and ND5 haplotypes recorded among *Ae. albopictus* from Cameroon.

COI						ND5				
Haplotype^a^	N	40	160	223	322	Haplotype^a^	N	93	237	297
REF [AJ971015]		C	C	A	A	REF [AJ971026]		T	A	T
H1 [JF309317]	133	T	.	.	G	H1 [JF309321]	132	.	.	.
H2 [JF309318]	4	.	.	.	G	H2 [JF309322]	18	.	.	C
H3 [JF309319]	13	.	.	G	G	H3 [JF309323]	1	A	.	.
H4 [JF309320]	3	.	T	.	G	H4 [JF309324]	2	.	G	.

Only polymorphic positions are shown, and are numbered with reference to the published *Ae. albopictus* sequences for COI (AJ971015; Reunion Island) and ND5 (AJ971026; Reunion Island). Dots represent identity with respect to reference. N: number of times the haplotype was found in the total sample.

a GenBank accession number in brackets.

**Table 5:** Summary statistics for mtDNA gene polymorphism in *Ae. albopictus* from Cameroon.

Sample	mtDNA gene	*N*	*Hp*	*S*	*HpD*	*π*	*K*	*D*	D*	F*	*Fs*
**Abong-Mbang**	ND5	10	2	1	0.53	0.002	0.53	1.30	0.80	1.03	1.03
	COI	10	2	2	0.53	0.003	1.07	1.64	1.03	1.31	2.34
**Bertoua**	ND5	10	3	2	0.64	0.002	0.73	0.12	−0.28	−0.20	−0.10
	COI	10	3	2	0.64	0.003	1.01	1.74	1.03	1.34	0.64
**Garoua-Boulai**	ND5	25	1	0	0.00	0.000	NC	NC	NC	NC	NC
	COI	25	1	0	0.00	0.000	NC	NC	NC	NC	NC
**Ayos**	ND5	10	2	1	0.20	0.001	0.20	−1.11	−1.24	−1.35	−0.34
	COI	10	3	2	0.38	0.001	0.56	−0.69	−0.28	−0.42	−0.59
**Bafia**	ND5	9	1	0	0.00	0.000	NC	NC	NC	NC	NC
	COI	9	1	0	0.00	0.000	NC	NC	NC	NC	NC
**Yaoundé**	ND5	10	2	1	0.20	0.001	0.20	−1.11	−1.24	−0.35	−0.34
	COI	10	2	1	0.20	0.001	0.20	−1.11	−1.24	−1.35	−0.34
**Buea**	ND5	28	3	2	0.26	0.001	0.27	−0.99	−0.91	−0.91	−1.13
	COI	29	3	2	0.13	0.001	0.20	−1.25	−0.73	−1.01	−1.63
**Douala**	ND5	9	2	1	0.39	0.001	0.39	0.16	0.84	0.75	0.48
	COI	9	2	2	0.39	0.002	0.78	0.20	1.06	0.95	1.59
**Pouma**	ND5	10	2	1	0.47	0.001	0.47	0.82	0.80	0.90	0.82
	COI	10	2	2	0.47	0.002	0.93	1.03	1.03	1.15	1.15
**Bamenda**	ND5	11	1	0	0.00	0.000	NC	NC	NC	NC	NC
	COI	10	1	0	0.00	0.000	NC	NC	NC	NC	NC
**Banganté**	ND5	11	2	1	0.18	0.001	0.18	−1.13	−1.29	−1.40	−0.41
	COI	11	2	2	0.18	0.001	0.36	−1.43	−1.66	−1.80	0.51
**Bafoussam**	ND5	10	1	0	0.00	0.001	NC	NC	NC	NC	NC
	COI	10	1	0	0.00	0.000	NC	NC	NC	NC	NC
**Overall**	ND5	153	4	3	0.24	0.001	0.25	−0.90	−0.68	−0.88	−1.83
	COI	153	4	3	0.24	0.001	0.42	−0.35	0.79	0.50	−0.56

N, number of sequences analyzed; Hp, number of haplotypes, S, number of segregating sites; HpD, haplotype diversity; π, nucleotide diversity; K, average number of nucleotide differences; D, Tajima's statistic [44]; D* and F*, Fu and Li's statistics [45]; Fs statistic [46]; no test is statistically significant; NC, not computed.

**Figure 3:**
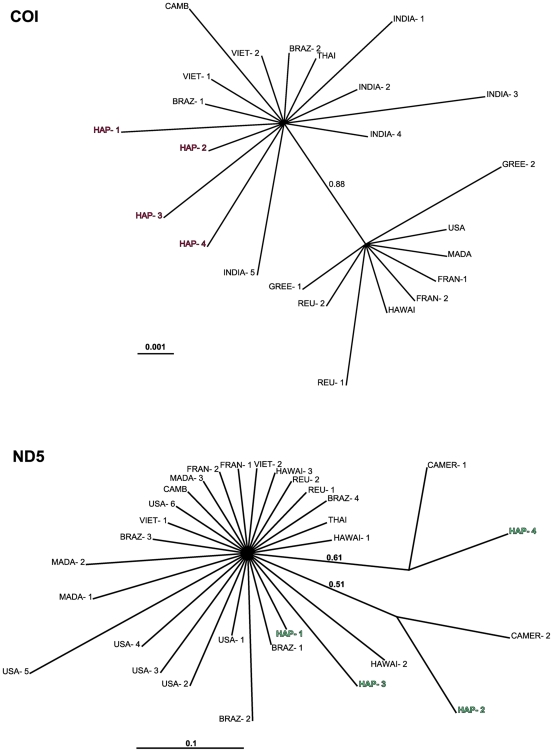
Bayesian inference hypothesis of *Ae. albopictus* phylogeny based on COI and ND5 sequence data. The phylogeny was constructed using MrBayes 3.1.2, ngen = 2 000 000. Best-fitting models selected using MRmodeltest (AIC) were HKY for COI and HKY+I+G for the ND5 nucleotide dataset. Branch support is indicated by the posterior probability values. Accession numbers of COI and ND5 outgroup sequences are given in supporting information file Table S2.

In order to determine the geographic origin of *Ae. albopictus* populations invading Cameroon, phylogenetic relationships between COI and ND5 sequences recorded in Cameroon and previously published sequences were assessed by using Bayesian inference. COI sequences segregated into two lineages (Figure 3). The first lineage encompassed specimens from temperate and subtropical areas (France, Greece, USA, Reunion Island and Madagascar) and the second encompassed specimens from tropical areas (Brazil, India, Cambodia, Thailand and Vietnam), including all Cameroonian specimens. Interestingly, a SNP at position 322 (Table 4) distinguished all tropical populations (G at 322) from all temperate/subtropical populations (A at 322). All the sequences were monophyletic at ND5. The sequence of the most frequent haplotype, H1, perfectly matched a cosmopolitan haplotype previously described in South-East Asia (Thailand, Cambodia, Vietnam), the Indian Ocean (Reunion Island and Madagascar), Europe (France), Hawaï and the USA [21], [22]. Haplotypes H2 and H4 have previously been described in Cameroon [51], whereas H3 is new.

## Discussion

This is the first study of the genetic variation and differentiation of *Ae. albopictus* populations invading continental Africa. Twelve mosquito population samples from Cameroon were analyzed using a set of six microsatellite DNA markers and two mtDNA genes (COI and ND5).

The set of microsatellite makers used here had already been tested on a single *Ae. albopictus* population from Indonesia [27]. The number of alleles detected in our study of a larger number of field populations was significantly higher at all loci, except for AaelB51. Overall, the observed level of polymorphism and the small number of deviations from Hardy-Weinberg equilibrium across markers and samples suggest that this set of microsatellite markers is suitable for population genetic studies of *Ae. albopictus* in Africa.

MtDNA markers have been extensively used to assess the genetic diversity of *Ae. albopictus* populations across most of its geographic range. The level of polymorphism found here within COI and ND5 sequences was low (four haplotypes for each gene) and consistent with previous studies of populations sampled in newly invaded areas [21], [22], [51], [52], in which the number of haplotypes per country never exceeded five, whatever the mtDNA marker used (ND5, COI or Cytb). The discrepancy in the levels of polymorphism obtained with the microsatellite and mtDNA markers was partly attributable to differences in mutation rates between maker classes. Microsatellites exhibit extreme intraspecific variability, Mendelian inheritance and high mutation rates, whereas mtDNA coding regions are relatively stable, haploidic and maternally inherited [53]. In addition, the mosquito samples we used for sequencing were small (10 individuals per sample) compared to those used for microsatellite genotyping. Even with larger numbers of sequenced specimens (27 and 40, in Garoua-Boulai and Buea respectively), the haplotype diversity indexes remained similar to those observed in other populations. This suggests that the low mtDNA diversity observed in *Ae. albopictus* in Cameroon was not related to the sample size. This low overall level of genetic diversity is consistent with recent introduction of *Ae. albopictus* in Cameroon.

The pattern of pairwise genetic differentiation estimated from the microsatellite data indicated a significant level of genetic structure among *Ae. albopictus* populations in Cameroon. No obvious relation between genetic and geographic distances was found, suggesting that the genetic structure has been shaped by additional biotic or abiotic factors. The Bayesian cluster analysis (i.e. existence of 2 genetic clusters co-occuring in most of cameroonian samples) supports a multiple introduction hypothesis. Recent studies have shown the important role of multiple introductions in successful colonization by invasive species [54] and in their genetic structure [55]. The introduction phase is usually subject to severe bottlenecks that reduce the genetic variability of founding populations to levels incompatible with an expansion phase. It is now thought that multiple introductions may help to reach sufficient additive genetic variability for efficient expansion and adaptation to novel environments. The Buea population was highly differentiated from other geographic samples, possibly reflecting distinct introduction events with different geographic origins. Indeed, Buea is geographically and culturally close to Nigeria, where the first waves of *Ae. albopictus* colonization in Africa were recorded in the 1990s [8]. Intensive trade between Buea and Nigeria may have favored passive dispersion of *Ae. albopictus* through accidental transportation of eggs, larvae or adults. Further analyses including samples from Nigeria would help to elucidate the relationships between Nigerian and Cameroonian populations of *Ae. albopictus*. Besides the effect of multiple introductions on the genetic structure of *Ae. albopictus* in Cameroon, the role of genetic drift must be considered. Indeed, the genetic variation observed in local populations of *Ae. albopictus* in Brazil [56] and in Reunion Island [57] has been attributed to genetic drift associated with restricted gene flow in recently established populations.

Analyses of COI sequences revealed that Cameroonian *Ae. albopictus* were related to specimens originating from tropical rather than temperate or subtropical areas. Unfortunately, we were unable to determine the geographic origin more precisely. Several studies have shown that *Ae. albopictus* populations that invaded Brazil [21], [22] were genetically related to tropical rather than temperate Asian populations. These results, together with our findings, suggest that tropical lineages possess genetic and physiological traits that allow them to colonize new tropical areas more efficiently than temperate lineages. Among these putative physiological differences between temperate and tropical populations, Hawley et al. [58] has reported distinct winter diapause behavior in laboratory conditions.

Despite the low genetic variation across the sampled *Ae. albopictus* populations, this study suggests that *Ae. albopictus* invasion of Cameroon involved multiple introductions from tropical sources. Further studies with more extensive sampling and a larger number of genetic markers, including other nuclear loci, are needed to confirm this hypothesis and to determine the precise geographic origin of Cameroonian *Ae. albopictus*.

## Supporting Information

Figure S1 **Microsatellite based Bayesian cluster analysis using STRUCTURE** [**41**] **.** (**A**). Estimates of Δ *K*, based on the second order rate of change of the likelihood function with respect to *K* [42], to determine the most likely number of clusters (*K*) in the data set. (**B**). Graphical representation of the data set for the most likely *K = 2*, where each color corresponds to a suggested cluster and each individual is represented by a vertical bar. The numbers in the x-axis correspond to a specific sample: 1) Abong-Mbang, 2) Bertoua, 3) Garoua-Boulai, 4) Ayos, 5) Bafia, 6) Yaoundé, 7) Buea, 8) Douala, 9) Pouma, 10) Bamenda, 11) Banganté and 12) Bafoussam.
(TIF)

Table S1 **Estimates of null allele frequencies per locus and per sample of** ***Ae. albopictus*** **.** Bolded: loci showing significant (P<0.05) heterozygote deficiency after correction for multiple testing.
(DOC)

Table S2 **Accession numbers of COI and ND5 sequences of outgroup specimens used for phylogenetic analysis.**
(DOC)

## References

[1] Kow CY, Koon LL, Pang FY (2001). Detection of Dengue Viruses in Field Caught Male *Aedes aegypti* and *Aedes albopictus* (Diptera: Culicidae) in Singapore by Type-Specific PCR.. J Med Entomol.

[2] Reiter P, Fontenille D, Paupy C (2006). *Aedes albopictus* as an epidemic vector of chikungunya virus: another emerging problem?. Lancet Infect Dis.

[3] Paupy C, Ollomo B, Kamgang B, Moutailler S, Rousset D (2010). Role of *Ae. albopictus* versus *Ae. aegypti* in dengue and chikungunya emergences in Central Africa: Laboratory and field evidence.. Vector Borne Zoonotic Dis.

[4] Smith CEG (1956). The history of dengue in tropical Asia and its probable relationship to the mosquitoes *Aedes aegypti*.. Journal of Tropical Medicine and Hygiene.

[5] Gratz NG (2004). Critical review of the vector status of *Aedes albopictus*.. Med Vet Entomol.

[6] Reiter P (1998). *Aedes albopictus* and the world trade in used tires, 1988–1995: the shape of things to come?. J Am Mosq Control Assoc.

[7] Cornel AJ, Hunt RH (1991). *Aedes albopictus* in Africa? First records of live specimens in imported tires in Cape Town.. J Am Mosq Control Assoc.

[8] Savage HM, Ezike VI, Nwankwo ACN, Spiegel R, Miller BR (1992). First record of breeding populations of *Aedes albopictus* in continental Africa: implications for arboviral transmission.. J Am Mosq Control Assoc.

[9] Fontenille D, Toto JC (2001). *Aedes (Stegomyia) albopictus* (Skuse), a potential new dengue vector in Southern Cameroon.. Emerg Infect Dis.

[10] Simard F, Nchoutpouen E, Toto JC, Fontenille D (2005). Geographic distribution and breeding site preference of *Aedes albopictus* and *Aedes aegypti* (Diptera: Culicidae) in Cameroon, Central Africa.. J Med Entomol.

[11] Kamgang B, Happi JY, Boisier P, Njiokou F, Hervé JP (2010). Geographic and ecological distribution of the dengue and chikungunya virus vectors *Aedes aegypti* and *Aedes albopictus* in three major Cameroonian towns.. Med Vet Entomol.

[12] Peyrefitte CN, Rousset D, Pastorino BA, Pouillot R, Bessaud M (2007). Chikungunya virus, Cameroon, 2006.. Emerging Infectious Diseases.

[13] Leroy EM, Nkoghe D, Ollomo B, Nze-Nkogue C, Becquart P (2009). Concurrent chikungunya and dengue virus infections during simultaneous outbreaks, Gabon.. Emerg Infect Dis.

[14] Dalla Pozza G, Majori G (1992). First record of *Aedes albopictus* establishment in Italy.. J Am Mosq Control Assoc.

[15] Hawley AH (1988). The biology of *Aedes albopictus*.. J Am Mosq Control Assoc.

[16] Scholte EJ, Schaffner F, Takken W, Knols BGJ (2007). Waiting the tiger: establishment and spread of the *Aedes albopictus* mosquito in Europe.. Emerging Pests and Vector-Borne Diseases in Europe.

[17] Delatte H, Desvars A, Bouétard A, Bord S, Gimonneau G (2010). Blood-feeding behavior of Aedes albopictus, a vector of Chikungunya on La Réunion.. Vector Borne Zoonotic Dis.

[18] Gubler DJ (2003). *Aedes albopictus* in Africa.. The Lancet Infect Dis.

[19] Kambhampati S, Black WC, Rai KS (1991). Geographic origin of the US and Brazilian *Aedes albopictus* inferred from allozyme analysis.. Heredity.

[20] Urbanelli S, Bellini R, Carrieri M, Sallicandro P, Celli G (2000). Population structure of *Aedes albopictus* (Skuse): the mosquito which is colonizing Mediterranean countries.. Heredity.

[21] Birungi J, Munstermann LE (2002). Genetic structure of *Aedes albopictus* (Diptera: Culicidae) populations based on mitochondrial ND5 sequences: evidence for an independent invasion into Brazil and United States.. Ann Entomol Soc Am.

[22] Mousson L, Dauga C, Garrigues T, Schaffner F, Vazeille M (2005). Phylogeography of *Aedes (Stegomyia) aegypti* (L.) and *Aedes (Stegomyia) albopictus* (Skuse) (Diptera: Culicidae) based on mitochondrial DNA variations.. Genet Res.

[23] Gubler DJ, Rosen L (1976). Variation among geographic strains of *Aedes albopictus* in susceptibility to infection with dengue viruses.. Am J Trop Med Hyg.

[24] Tesh RB, Gubler DJ, Rosen L (1976). Variation among geographic strains of *Ae. albopictus* in susceptibility to infection with chikungunya virus.. Am J Trop Med Hyg.

[25] Lourenço-de-Oliveira R, Vazeille M, Bispo-de-Filippis AM, Failloux AB (2003). Large genetic differentiation and low variation in vector competence for dengue and yellow fever viruses of *Aedes albopictus* from Brazil, the United States, and the Cayman islands.. Am J Trop Med Hyg.

[26] Morlais I, Ponçon N, Simard F, Cohuet A, Fontenille D (2004). Intraspecific nucleotide variation in An. gambiae promises new insights into the biology of malaria vectors.. Am J Trop Med Hyg.

[27] Porreta D, Gargani M, Bellini R, Calviti M, Urbanelli S (2006). Isolation of microsatellite markers in the tiger mosquito *Aedes albopictus* (Skuse).. Mol Ecol Notes.

[28] El Mousadik A, Petit RJ (1996). High level of genetic differentiation for allelic richness among populations of the argan tree (*Argania spinosa* L. Skeels) endemic of Morocco.. Theor Appl Genet.

[29] Nei M (1987). Molecular evolutionary genetics..

[30] Goudet J (1995). FSTAT (version 1.2): a computer software to calculate F-statistics.. J Heredity.

[31] Belkhir K, Borsa P, Chikhi L, Raufaste N, Bonhomme F (2004). GENETIX 4.05, logiciel sous Windows TM pour la génétique des populations.. http://www.genetix.univmontp2.fr/genetix/genetix.htm.

[32] Raymond M, Rousset F (1995). GENEPOP (version 1.2): population genetics software for exact tests and ecumenicism.. J Hered.

[33] Weir BS, Cockerham CC (1984). Estimating F-statistics for the analysis of population structure.. Evolution.

[34] Holm S (1979). A simple sequentially rejective multiple test procedure.. Scand J Stat.

[35] Van Oosterhout C, Hutchinson WF, Wills DP, Shipley P (2004). MICROCHECKER: Software for identifying and correcting genotyping errors in microsatellite data.. Mol Ecol.

[36] Rousset F (1997). Genetic differentiation and estimation of gene flow from F-statistics under isolation by distance.. Genetics.

[37] Liu K, Muse SV (2005). POWERMARKER: Integrated analysis environment for genetic marker data.. Bioinformatics.

[38] Nei M, Nei M, Koehn R (1983). Genetic polymorphism and the role of mutation in evolution.. Evolution of genes and proteins.

[39] Felsenstein J (1989). PHYLIP-phylogeny inference package (version 3.2).. Cladistics.

[40] Page RDM (1996). TREEVIEW: An application to display phylogenetic trees on personal computers.. Comput Appl Biosci.

[41] Pritchard JK, Stephens M, Donnelly P (2000). Inference of population structure using multilocus genotype data.. Genetics.

[42] Evanno G, Regnaut S, Goudet J (2005). Detecting the number of clusters of individuals using the software STRUCTURE: a simulation study.. Mol Ecol.

[43] Bonacum J, DeSalle R, O'Grady P, Olivera D, Wintermute J (2001). New nuclear and mitochondrial primers for systematics and comparative genomics in Drosophilidae.. Dros Inf Serv.

[44] Thompson JD, Higgins D, Gibson TJ (1994). CLUSTAL W: improving the sensitivity of progressive multiple sequence alignment through sequence weighting, position-specific gap penalties and weight matrix choice.. Nucleic Acids Res.

[45] Rozas J, Sanchez-DelBarrio JC, Messeguer X, Rozas R (2003). DnaSP, DNA polymorphism analyses by the coalescent and other methods.. Bioinformatics.

[46] Tajima F (1989). The effect of change in population size on DNA polymorphism.. Genetics.

[47] Fu YX, Li WH (1993). Statistical tests of neutrality mutations.. Genetics.

[48] Fu YX (1997). Statistical tests of neutrality of mutations against population growth, hitchhiking and background selection.. Genetics.

[49] Nylander JAA (2004). MrModeltest v2..

[50] Ronquist F, Huelsenbeck JP (2003). MRBAYES v3: Bayesian phylogenetic inference under mixed models.. Bioinformatics.

[51] Usmani-Brown S, Cohnstaedt L, Munstermann EL (2009). Population Genetics of *Aedes albopictus* (Diptera: Culicidae) Invading Populations, Using Mitochondrial nicotinamide Adenine Dinucleotide Dehydrogenase Subunit 5 Sequences.. Ann Entomol Soc Am.

[52] Maia RT, Scarpassa VM, Maciel-Litaiff LH, Tadei WP (2009). Reduced levels of genetic variation in *Aedes albopictus* (Diptera: Culicidae) from Manaus, Amazonas State, Brazil, based on analysis of the mitochondrial DNA *ND5* gene.. Gen Mol Res.

[53] Mas-Coma S, Bargues MD (2009). Populations, hybrids and the systematic concepts of species and subspecies in Chagas disease triatomine vectors inferred from nuclear ribosomal and mitochondrial DNA.. Acta Tropica.

[54] Kolbe JJ, Glor RE, Schettino LR, Lara AC, Larson A (2004). Genetic variation increases during biological invasion by a Cuban lizard.. Nature.

[55] Zalewski A, Michalska-Parda A, Bartoszewicz M, Kozakiewicz M, Brzeziński M (2010). Multiple introductions determine the genetic structure of an invasive species population: American mink *Neovison vison* in Poland.. Biol Conserv.

[56] Ayres CF, Romao TP, Melo-Santos MA, Furtado AF (2002). Genetic diversity in Brazilian populations of *Aedes albopictus*.. Mem Inst Oswaldo Cruz.

[57] Paupy C, Girod R, Salvan M, Rodhain F, Failloux AB (2001). Population structure of *Aedes albopictus* from La Réunion Island (Indian Ocean) with respect to susceptibility to a dengue virus.. Heredity.

[58] Hawley WA, Reiter P, Copeland RS, Pumpuni CB, Craig GB (1987). *Aedes albopictus* in North America: probable introduction in used tires from northern Asia.. Science.

