# Supplementary figures and images for: Genetic Structure of the Tiger Mosquito, Aedes albopictus, in Cameroon (Central Africa)
Source: PLoS One. 2011 May 24;6(5):e20257. doi: 10.1371/journal.pone.0020257 (PMC3101236; doi:10.1371/journal.pone.0020257)

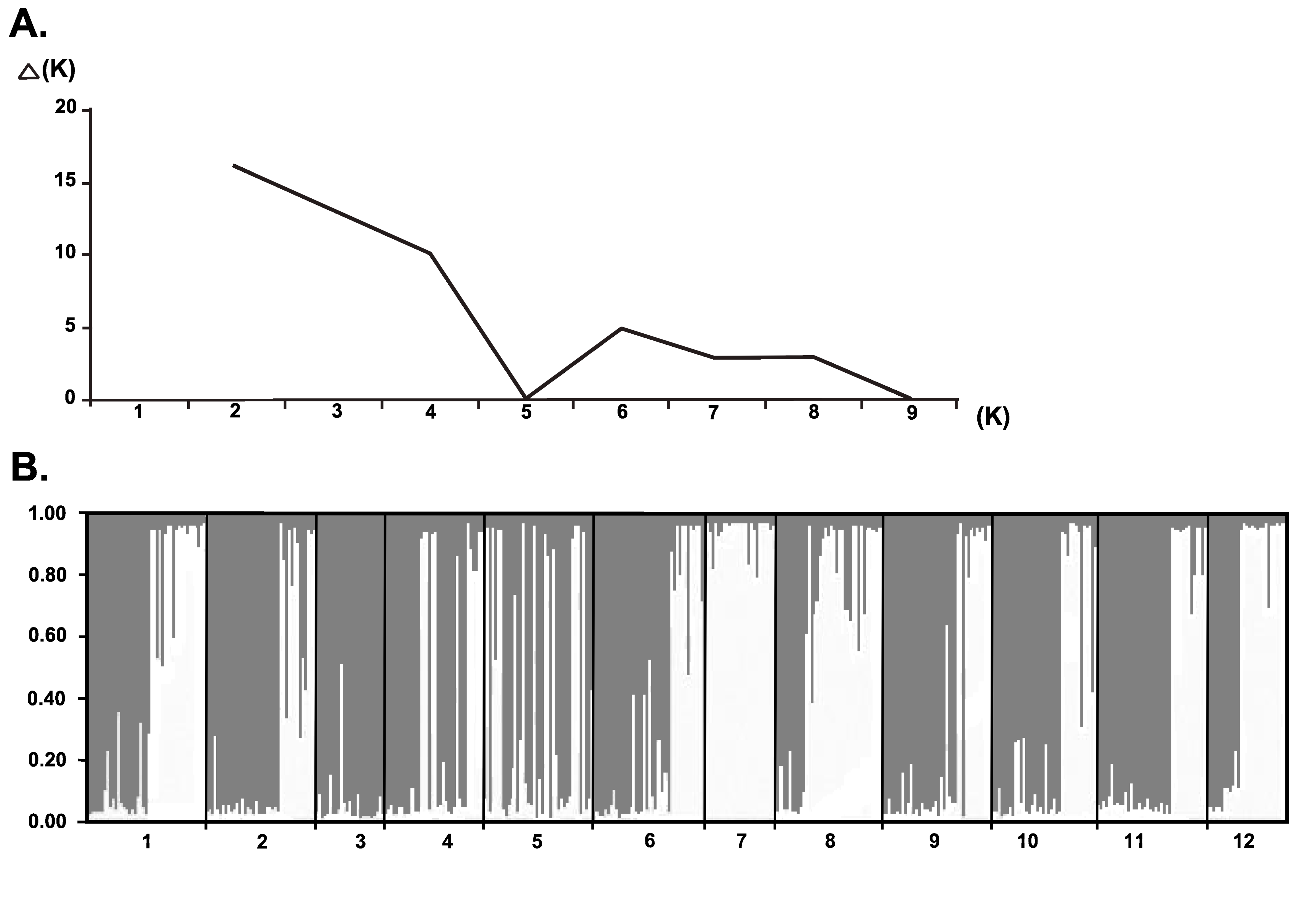

Supplement: Figure S1 — Microsatellite based Bayesian cluster analysis using STRUCTURE [41] . (A). Estimates of Δ K, based on the second order rate of change of the likelihood function with respect to K [42], to determine the most likely number of clusters (K) in the data set. (B). Graphical representation of the data set for the most likely K = 2, where each color corresponds to a suggested cluster and each individual is represented by a vertical bar. The numbers in the x-axis correspond to a specific sample: 1) Abong-Mbang, 2) Bertoua, 3) Garoua-Boulai, 4) Ayos, 5) Bafia, 6) Yaoundé, 7) Buea, 8) Douala, 9) Pouma, 10) Bamenda, 11) Banganté and 12) Bafoussam. (TIF) [file pone.0020257.s001.tif]
