# Supplementary material for: Genetic Structure of the Tiger Mosquito, Aedes albopictus, in Cameroon (Central Africa)
Source: PLoS One. 2011 May 24;6(5):e20257. doi: 10.1371/journal.pone.0020257 (PMC3101236; doi:10.1371/journal.pone.0020257)
Supplement: Table S1 — Estimates of null allele frequencies per locus and per sample of Ae. albopictus . Bolded: loci showing significant (P<0.05) heterozygote deficiency after correction for multiple testing. (DOC) [file pone.0020257.s002.doc]

| **Sample** | **AealbB6** | **AealbD2** | **AealbA9** | **AealbB51** | **AealbB52** | **AealbF3** |
| --- | --- | --- | --- | --- | --- | --- |
| **Abong-Mbang** | **0.170** | 0.000 | 0.043 | **-** | 0.000 | 0.000 |
| **Bertoua** | **0.201** | 0.016 | 0.000 | 0.1601 | 0.0263 | 0.000 |
| **Garoua-boulaï** | 0.068 | 0.028 | 0.000 | **-** | 0.0859 | 0.000 |
| **Ayos** | **0.129** | 0.008 | 0.000 | 0.000 | 0.2236 | 0.0019 |
| **Bafia** | **0.239** | 0.066 | 0.000 | 0.000 | 0.000 | 0.000 |
| **Yaoundé** | 0.013 | 0.055 | 0.069 | 0.000 | 0.174 | 0.000 |
| **Buea** | 0.050 | 0.000 | 0.078 | **-** | 0.1526 | 0.000 |
| **Douala** | 0.000 | 0.074 | 0.000 | 0.000 | 0.000 | 0.000 |
| **Pouma** | 0.082 | 0.000 | 0.015 | **0.274** | 0.000 | 0.000 |
| **Bamenda** | **0.206** | 0.001 | 0.000 | **-** | 0.000 | 0.000 |
| **Banganté** | **0.187** | 0.100 | 0.000 | 0.000 | 0.0364 | 0.000 |
| **Bafoussam** | **0.138** | 0.000 | 0.000 | **-** | 0.1358 | 0.000 |
