# Supplementary material for: Genetic Structure of the Tiger Mosquito, Aedes albopictus, in Cameroon (Central Africa)
Source: PLoS One. 2011 May 24;6(5):e20257. doi: 10.1371/journal.pone.0020257 (PMC3101236; doi:10.1371/journal.pone.0020257)
Supplement: Table S2 — Accession numbers of COI and ND5 sequences of outgroup specimens used for phylogenetic analysis. (DOC) [file pone.0020257.s003.doc]

| **mtDNA Marker** | **Geographical Area** | **Outgroup Name** | **Country** | **Climate** | **Accession Number** |
| --- | --- | --- | --- | --- | --- |
| **COI** | **Europe, West** | FRAN-1 | France | Temperate | AJ971008 |
|  |  | FRAN-2 | France | Temperate | AJ971009 |
|  |  | GREE-1 | Greece | Temperate | AY748238 |
|  |  | GREE-2 | Greece | Temperate | AY748239 |
|  | **America, North** | USA | United States of America | Temperate | AJ971005 |
|  | **Pacific Ocean, North** | HAWAI | Hawaï (USA) | Sub-Tropical | AJ971011 |
|  | **Indian Ocean, South West** | MADA | Madagascar | Sub-Tropical | AJ971007 |
|  |  | REU-1 | La Réunion Island | Sub-Tropical | AJ971012 |
|  |  | REU-2 | La Réunion Island | Sub-Tropical | AJ971013 |
|  | **Asia, South** | INDIA-1 | India | Tropical | AY729984 |
|  |  | INDIA-2 | India | Tropical | AY834241 |
|  |  | INDIA-3 | India | Tropical | DQ310142 |
|  |  | INDIA-4 | India | Tropical | DQ424959 |
|  |  | INDIA-5 | India | Tropical | EU250306 |
|  | **America, South** | BRAZ-1 | Brazil | Tropical | AJ971003 |
|  |  | BRAZ-2 | Brazil | Tropical | AJ971014 |
|  | **Asia, South-East** | CAMB | Cambodia | Tropical | AJ971006 |
|  |  | THAI | Thaïland | Tropical | AJ971015 |
|  |  | VIET-1 | Vietnam | Tropical | AJ971004 |
|  |  | VIET-2 | Vietnam | Tropical | AJ971010 |
| **ND5** | **Europe, West** | FRAN | France | Temperate | AJ971022 |
|  | **America, North** | USA-1 | United States of America | Temperate | AY049970 |
|  |  | USA-2 | United States of America | Temperate | AY049972 |
|  |  | USA-3 | United States of America | Temperate | AY049971 |
|  |  | USA-4 | United States of America | Temperate | AY049973 |
|  |  | USA-5 | United States of America | Temperate | AY049974 |
|  |  | USA-6 | United States of America | Temperate | AJ971018 |
|  | **Pacific Ocean, North** | HAWAI-1 | Hawaï (USA) | Sub-Tropical | EU118296 |
|  |  | HAWAI-2 | Hawaï (USA) | Sub-Tropical | EU118297 |
|  | **Indian Ocean, South West** | MADA-1 | Madagascar | Sub-Tropical | AY049976 |
|  |  | MADA-2 | Madagascar | Sub-Tropical | AY049975 |
|  |  | MADA-3 | Madagascar | Sub-Tropical | AJ971020 |
|  |  | REU-1 | La Réunion Island | Sub-Tropical | AY785426 |
|  |  | REU-2 | La Réunion Island | Sub-Tropical | AY785425 |
|  | **Africa, Central** | CAMER-1 | Cameroon | Tropical | EU118294 |
|  |  | CAMER2 | Cameroon | Tropical | EU118295 |
|  | **America, South** | BRAZ-1 | Brazil | Tropical | AY049968 |
|  |  | BRAZ-2 | Brazil | Tropical | AY049969 |
|  |  | BRAZ-3 | Brazil | Tropical | AJ971016 |
|  |  | BRAZ-4 | Brazil | Tropical | AJ971028 |
|  | **Asia, South-East** | CAMB | Cambodia | Tropical | AJ971019 |
|  |  | THAI | Thaïland | Tropical | AJ971028 |
|  |  | VIET-1 | Vietnam | Tropical | AJ971017 |
|  |  | VIET-2 | Vietnam | Tropical | AJ971023 |
